# The improvement of deformability in AA7075 alloy through cryogenic treatment and its correlation with microstructural evolution and FE modelling

**DOI:** 10.1038/s41598-024-67518-4

**Published:** 2024-07-20

**Authors:** Suwaree Chankitmunkong, Dmitry G. Eskin, Ussadawut Patakham, Wares Chancharoen, Saran Seehanam, Chaowalit Limmaneevichitr, Phromphong Pandee

**Affiliations:** 1 Department of Industrial Engineering, School of Engineering, King Mongkut’s Institute of Technology Ladkrabang, Chalongkrung Road, Ladkrabang, Bangkok, 10520 Thailand; 2 Brunel University London, BCAST, Uxbridge, Middlesex, UB8 3PH UK; 3 National Metal and Materials Technology Center, National Science and Technology Development Agency, 114 Thailand Science Park, Klong Luang, Pathumthani 12120 Thailand; 4 Princess Srisavangavadhana College of Medicine, Chulabhorn Royal Academy, 906 Kamphaeng Phet 6 Rd, Talat Bang Khen, Lak Si, Bangkok, 10210 Thailand; 5 Department of Mechanical Engineering, Faculty of Engineering, King Mongkut’s University of Technology Thonburi, 126 Pracha-Uthit Road, Bang Mod, Thungkhru, Bangkok, 10140 Thailand; 6 Department of Production Engineering, Faculty of Engineering, King Mongkut’s University of Technology Thonburi, 126 Pracha-Uthit Road, Bang Mod, Thungkhru, Bangkok, 10140 Thailand; 7 Center for Lightweight Materials, Design and Manufacturing, King Mongkut’s University of Technology Thonburi, 126 Pracha-Uthit Road, Bang Mod, Thungkhru, Bangkok, 10140 Thailand

**Keywords:** Recrystallization, True stress-strain curves, Al_2_CuMg phase, Plastic Equivalent Strain (PEEQ), Metals and alloys, Mechanical engineering

## Abstract

Cryogenic treatment has high potential for improving the deformation behavior through the recrystallization at a low temperature. In this work, true stress–strain curves were obtained via compression tests to understand the deformation behavior of an AA7075 under cryogenic conditions. Results showed a significant improvement in the flow stress of AA7075, increasing from 260 to 560 MPa at the yield point. The strain hardening exponent (*n*) also increased from 0.25 to 0.35 after deformation at cryogenic temperatures. The presence of Al_2_CuMg phase influenced the deformation texture of the tested aluminum alloy, resulting in more elongated grains and fine sub-grains after deformation at cryogenic temperatures, due to the hindered recrystallization. Microstructure evolution after deformation at room and cryogenic temperatures was investigated using EBSD technique to characterize texture and recrystallized grains. The results indicated that the spacing of the high-angle grain boundaries (HAGBs) in the sample deformed at room temperature was slightly larger than in the cryogenically treated sample. The alloy deformed at the cryogenic temperature exhibited a higher strain hardening exponent (n = 0.35) compared to room temperature deformation (*n* = 0.25). Furthermore, finite element analysis supported the experimental findings, showing that the Plastic Equivalent Strain (PEEQ) of the model tested at cryogenic temperature was higher than at room temperature, attributed to grain refinement during low-temperature deformation. The calculated effective stress responses at cryogenic temperatures for the investigated flow stress aligned well with the experimental results. These new aspects and mechanisms of deformation of aluminum alloys at cryogenic temperatures can improve the formability of high-strength alloys in the future production of more complex and integrated lightweight components.

## Introduction

An AA7075 aluminum alloy is a precipitation-hardened alloy used in applications requiring high strength and toughness, good corrosion properties and wear resistance, i.e., in automotive and aerospace components^1^. However, due to its limited formability at room temperature, parts with complicated shapes cannot be manufactured through the traditional cold stamping or pressing of rolled sheets^2^. There have been a number of studies conducted to date that present several ways for enhancing the formability of aluminum alloys at high temperatures^2–4^. However, such high temperature procedures can cause negative effects such as unpredicted thinning and necking, as well as lower age-hardening potential. Therefore, various kinds of novel forming processes were developed to eliminate this deficiency. Previous research reported that the formability of AA7075-T6 can be improved through warm forming^4^. The enhancement of sheet forming formability is more obvious in processing materials with a lower strain hardening exponent but higher ductility^5^. However, it is not favorable for metal stamping as galling defects tend to occur between the aluminum specimen and the mold surface^6^. It was accepted that cryogenic forming of Al–Mg alloy improved strength, elongation, and forming limits, which offered opportunities for deep drawing of complex-shape products^7^. However, there is a limit to the study of the development of aluminum alloy properties for the fine blanking process. This process has been developed to eliminate secondary operations such as milling or grinding. Previous work determined the effect of process parameters, including clearance between punch and die, and application of V-ring indenter or flat blank holder on stress and strain distribution in the shearing zone to obtain a good quality surface^8^. Unfortunately, there has been little research into the microstructure change and deformation behavior of soft materials at cold temperatures, including aluminum and its alloys^9^. Generally, forming complex sheet aluminum products at room temperature often results in the development of galling defects^6^. A thick layer of compaction galling occurred after forming at higher temperature and led to the damage on surface of an aluminum specimen^6^. However, it was demonstrated that an Al–Mg–Si alloy treated under cryogenic temperature had an increased flow stress^10^, which may facilitate forming complex sheet aluminum products. This finding aligns with another study where cryogenic processing allowed for the forming of complex shapes in an AA5XXX-series alloy without occurrence of crack defects^7^. Previous studies of the formability of 7075-T6 aluminum alloy vehicle supports reported that the parts broke at 20 °C, while could be easily deformed at a high temperature^11,12^. This resulted from the fracture mode of this alloy changing from a ductile fracture mode to a quasi-brittle fracture when changing the deformed temperature from about 200 °C to a sub-zero temperature^13^.

Cryogenic treatment is a traditional and supplementary method to improve the performance of tool steels^14^. The technology was also recently suggested for low-temperature heat treatment of aluminum alloy components in order to improve their properties^10,15–18^. It was reported that the cryogenic treatment can improve the hardness, tensile strength, wear resistance and corrosion resistance of an aluminum alloy. Moreover, the cryogenic treatment removed tensile residual stresses and created small amount of compressive residual stresses^16,19^. Cryogenic treatment parameters such as cooling rate, heating rate, soaking temperature and soaking time are very important for improving material performance^20^. For the evaluation of deformability of high-strength steel and light-weight alloys, the strain hardening exponent (*n*) of materials is a key parameter that can be estimated to characterize how quickly the material gains strength when it is being deformed^21^. Previously, it was observed that the *n* value for aluminum alloy sheets initially increased before stabilizing with increasing strain in the plastic deformation region and a greater *n* value was obtained with a larger grain size^22^. Liu et al.^23^ also found that *n* was the largest for an AA5056 alloy that was subjected to solid solution treatment as compared to that after T4 heat treatment, which was attributed to the much lower shear stress for dislocation slip.

In order to investigate the possibility of employing a cryogenic treatment to enhance the mechanical properties of an AA7075 aluminum alloy, flow behavior and microstructure evolution were determined and discussed. In addition, the deformation behavior was investigated through compression tests and finite element modeling. The effects of cryogenic treatment on microstructure and mechanical properties are reported to provide guidance for the application of cryogenic treatment to successfully improve the formability of high-strength AA7075 aluminum alloy.

## Experimental method

An as-cast AA7075 plate of 170 mm × 150 mm × 17 mm was made by remelting from a commercial ingot with the chemical composition as shown in Table 1. To study the effect of cryogenic treatment on deformability, cylindrical samples were machined from an AA7075 cast plate. Each sample had a diameter of 10 mm and a height of 20 mm.

**Table 1:** Composition of the AA7075 aluminum alloy (wt%).

Alloys	Zn	Mg	Cu	Mn	Fe	Si	Cr
AA7075 cast ingot	5.38	2.42	1.62	0.06	0.34	0.16	0.21

The samples were ground with silicon carbide (SiC) abrasive papers and polished with diamond suspension. After that, the samples were anodized in a Barker solution at 20 VDC for 2 min. Microstructure of the as-cast alloy was observed by an Olympus optical microscope under normal and polarized light.

The compression tests were performed at room (Room-T) and cryogenic temperatures (Cryo-T) at a strain rate of 0.01 s^−1^ to obtain true stress–strain curves on a Gotech Al-7000-LA10 universal testing machine. The samples for cryogenic treatment were tested after being immersed in liquid nitrogen (− 196 °C) for 30 min. There were three specimens for each condition. The strain hardening exponent (*n* value) of materials is obtained from the slope of the true stress vs true strain curve in a compression test, plotted on a logarithmic scale. The relationship between the stress and the strain can be expressed as a following Eq.^24^:

1 $$\sigma =k{\varepsilon }^{n}$$

where $\sigma$ and $\varepsilon$ are the true stress and true strain, respectively, *k* is strength coefficient, and *n* is strain hardening coefficient.

In addition, the crystallographic texture and grain boundary evolution after compression testing at room and cryogenic temperatures were investigated. For crystal orientation analysis, the sample surfaces were first ground with SiC paper and polished to a 1 μm finish using a diamond suspension, and then the samples were polished using a vibratory polisher with colloidal silica in a 0.05 μm suspension. To characterize secondary phase precipitation, a field emission scanning electron microscope (FE-SEM) equipped with an energy dispersive X-ray spectrometer (EDS) (Apreo S Thermo Fisher Scientific) was used. The electron back scatter diffraction (EBSD) analysis was performed on a Hitachi SEM model S-3400N equipped with TSL-EDAX EBSD. The Ametek EDAX TSL OIM EBSD-software was used for automated recognition and indexing of EBSD. The maximum resolution of the orientation maps obtained with this equipment is estimated to be approximately 0.5–1 µm for aluminum.

## Results and discussion

### Microstructure of an alloy after casting and after the cryogenic treatment

**Figure 1:**
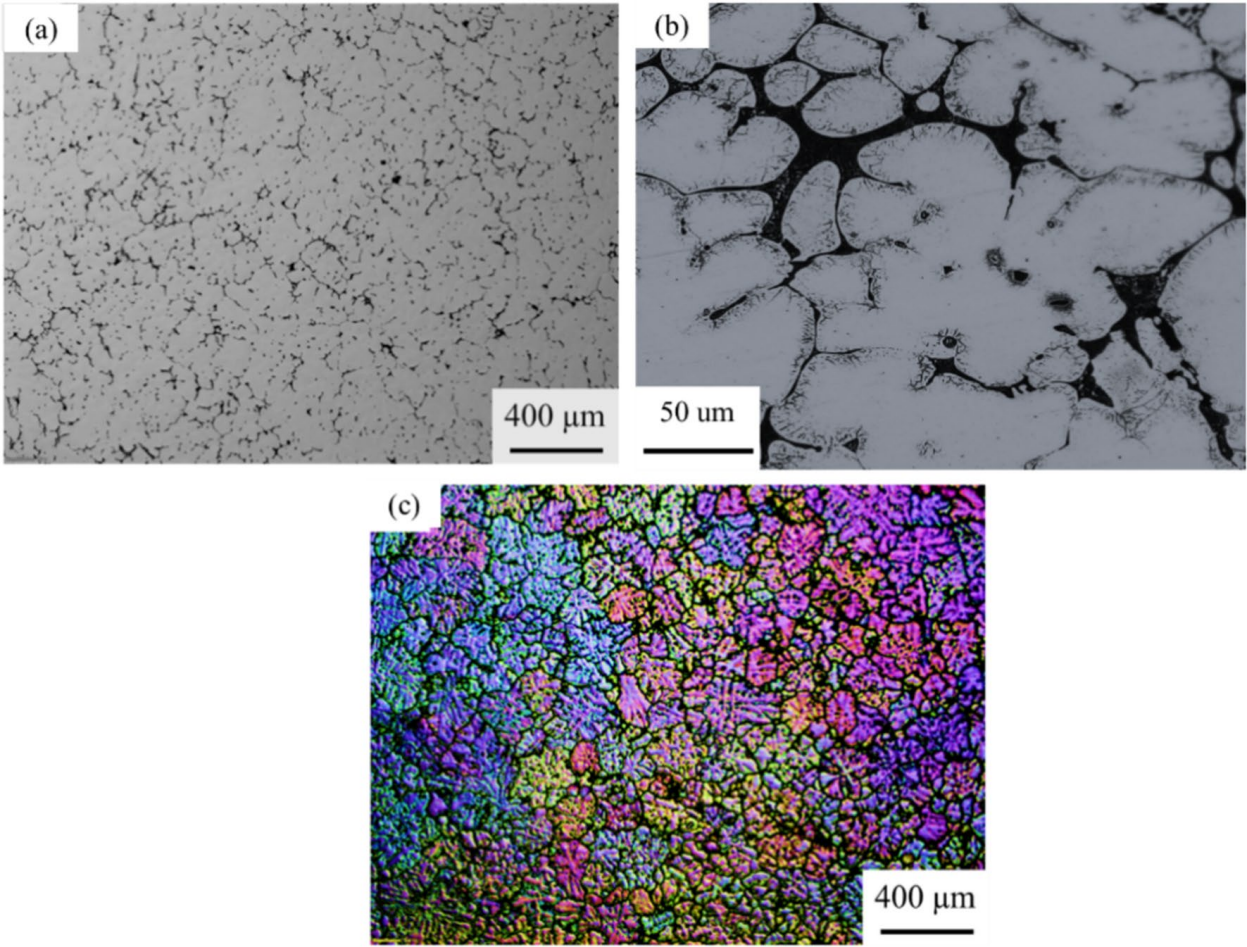
Microstructure of an as-cast AA7075 aluminum alloy: (**a**) low magnification, (**b**) high magnification, and (**c**) polarized light revealing grain structure.

Figure 1 shows the initial microstructure of an as-cast AA7075 aluminum alloy that comprises dendrites of the aluminum solid solution and divorced eutectic comprising Al_7_Cu_2_Fe, Al_2_CuMg, and MgZn_2_ constitutive phases at grain boundaries before the deformation process, similar to previous work^25^. The grain structure is shown in Fig. 1c with an average grain size of 297 ± 5 µm.

**Figure 2:**
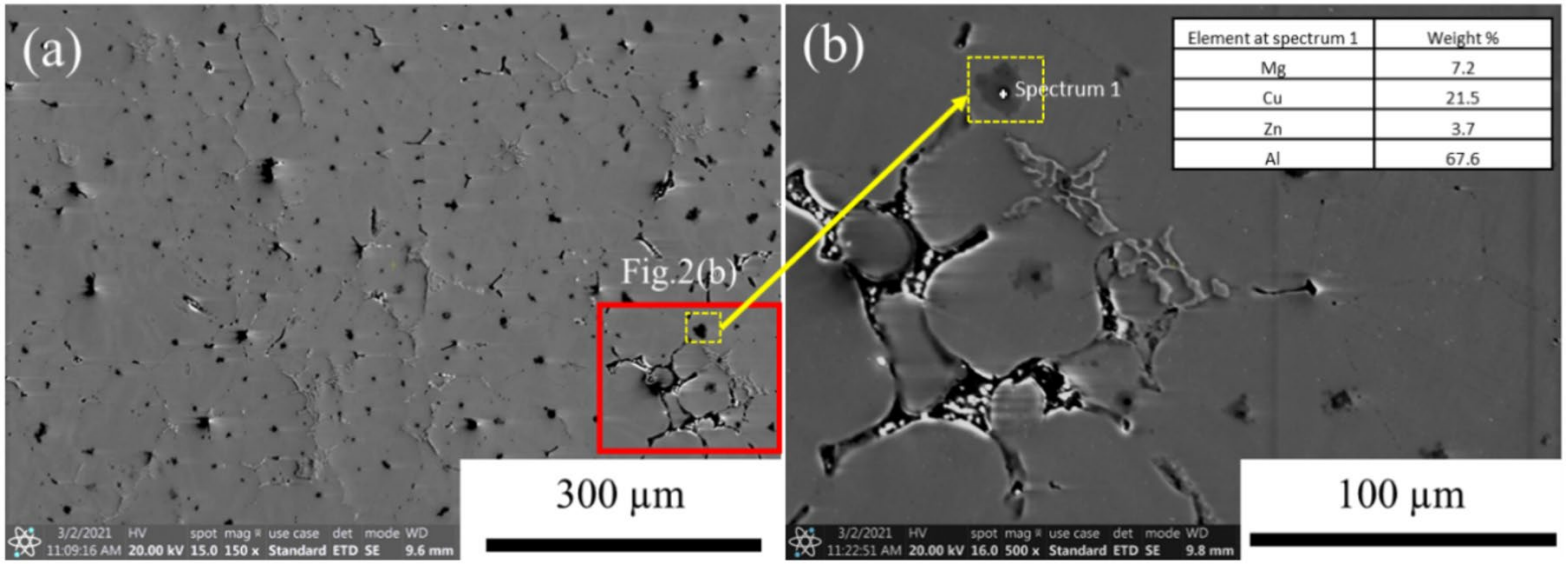
(**a**) The distribution of secondary phase particles in a cryogenically treated sample and (**b**) high magnification of the secondary phases with EDS analysis in the marked point.

Figure 2a shows the distribution of secondary phases in the sample that was treated at a cryogenic temperature. The results found that a secondary phase precipitated and dispersed in the Al-grain matrix after exposure to the cryogenic temperature. The EDS analysis result showed that the second phase contained four main elements of Al/Cu/Zn/Mg as can be seen in the inset table of EDS value of Fig. 2b. According to EDS results, it can be precalculated that this secondary phase could be the Al_2_CuMg phase, similar to previously reported findings^25^. It was previously reported that Cu-containing secondary phases can precipitate after deep cryogenic treatment of an AA7050 alloy^26^. The Al_2_CuMg precipitates can interact with grain boundaries, affecting their mobility and behavior during deformation processes such as grain boundary sliding, grain boundary migration, and grain boundary pinning. These interactions adversely affect the mechanical properties of the Al–Zn–Mg–Cu alloy^27^ and play important roles in the formation of recrystallized textures in aluminum^28^.

### Feasibility of deformation of an as-cast AA7075 alloy under cryogenic treatment

**Figure 3:**
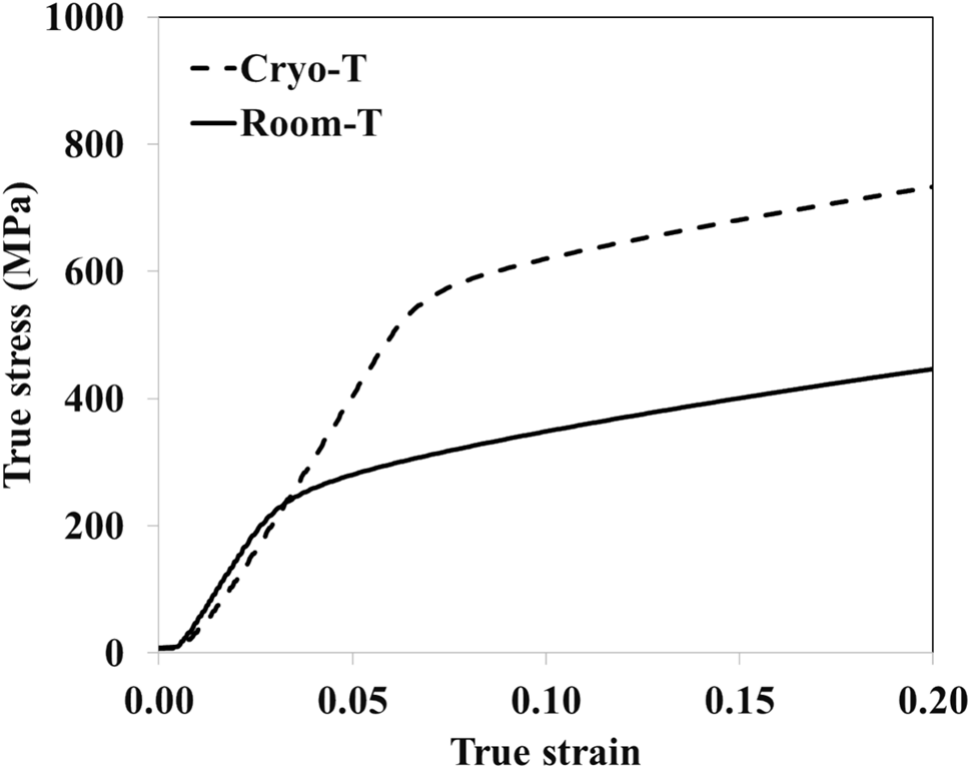
True stress–strain curves for an AA7075 aluminum alloy upon compression testing at a strain rate of 0.01 s^−1^ at room and cryogenic temperatures.

To estimate the feasibility of manufacturing complex stamping components from an AA7075 alloy by cryogenic forming, the deformation behavior was analyzed through the true stress–strain curves after compression testing at room and cryogenic temperatures as shown in Fig. 3.

In forming operation of ductile materials such as aluminum alloys, the stain hardening exponent is an important feature that can be estimated from true stress–strain curves (Fig. 3) to predict the deformation behavior, as shown in Table 2.

**Table 2:** Material properties of an AA7075 alloy after compression testing under room and cryogenic temperatures.

Condition testing	Flow stress (*σ*_*f*_) at yield (MPa)	Strain hardening exponent (*n*)	Strength coefficient (*k*)
Room temperature	250 ± 3	0.25 ± 0.01	353 ± 6
Cryogenic temperature	550 ± 7	0.35 ± 0.02	616 ± 8

One can see that the AA7075 alloy deformed at the cryogenic temperature had a higher strain hardening exponent (*n* = 0.35) as compared to that at room temperature (*n* = 0.25), which may lead to the good deformability of this aluminum alloy because it can be work-hardened sufficiently in critical areas to better distribute the strain over other areas, thus reducing local buildup of strain^29^. It was reported that work hardening is strongly dependent on temperature^30^. Moreover, the high strain hardening exponent can benefit the deformation behavior of aluminum that has poor resistance to seizure and galling because it easily adheres to the steel forming tool, leading to surface damage of both the tool and aluminum alloy^31,32^. In addition, it was reported that the increased strain hardening rate at cryogenic temperatures was linked with the reduced dynamic recovery (typically operated via cross-slip of screw dislocations)^33^. This is perfectly in line with the higher dislocation densities and proportion of screw dislocations upon low temperature deformation as reported elsewhere^33^.

**Figure 4:**
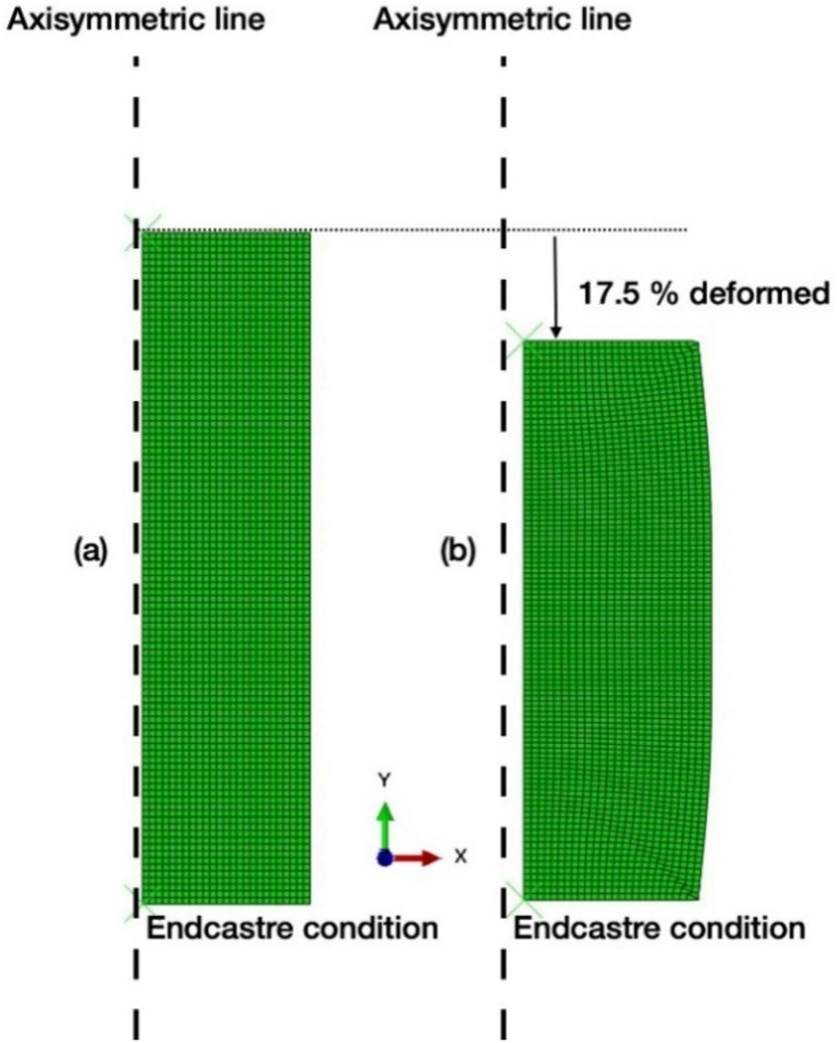
The initial geometry and conditions of finite element analysis was 50 mm × 200 mm with quadrilateral meshing (**a**, left). The final geometry and conditions of compression process was performed until 17.5% of deformation (**b**, right).

Finite element simulation was used to evaluate the deformation behavior of an AA7075 alloy at room and cryogenic temperatures by ABAQUS/CAE software using the standard elastic–plastic model. The input material properties were determined from true stress–strain curves similar to those shown in Fig. 3. In addition to the elastic–plastic stress–strain curve, Poisson’s ratio was set as 0.3 as well as the friction coefficient between the compressing tool and the specimen was 0.1. The model was 2D axis-symmetric 50 mm in width (x-axis) and 200 mm in height (y-axis) using the quadrilateral linear-order type of meshing as shown in Fig. 4a. The experimental data for compression at both room and cryogenic temperatures (Fig. 3) were used as material properties in the simulations, which was divided into two models, the room temperature model and the cryogenic temperature model. The strain rate was 0.01 mm per second downward to the negative y axis and was applied until 17.5% of deformation occurred in the finite element analysis model as shown in Fig. 4b to simulate the compression process. The bottom side of the model domain was applied as an endcastre boundary condition to fix all directions.

**Figure 5:**
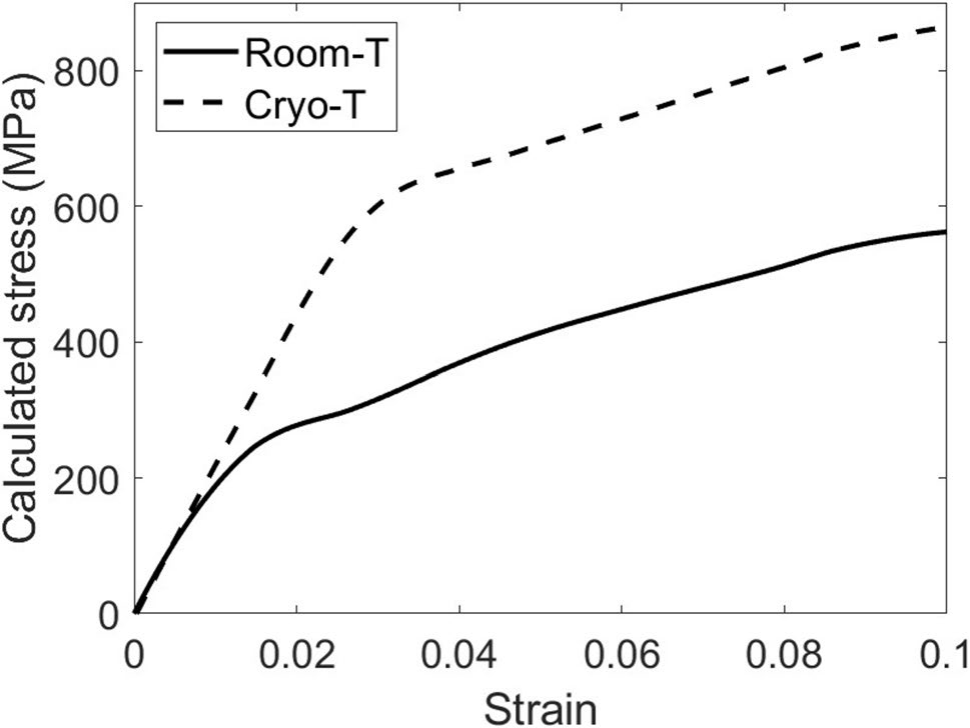
Calculated stress–strain curves using finite element model of the alloy deformed at room temperature and cryogenic temperature.

**Figure 6:**
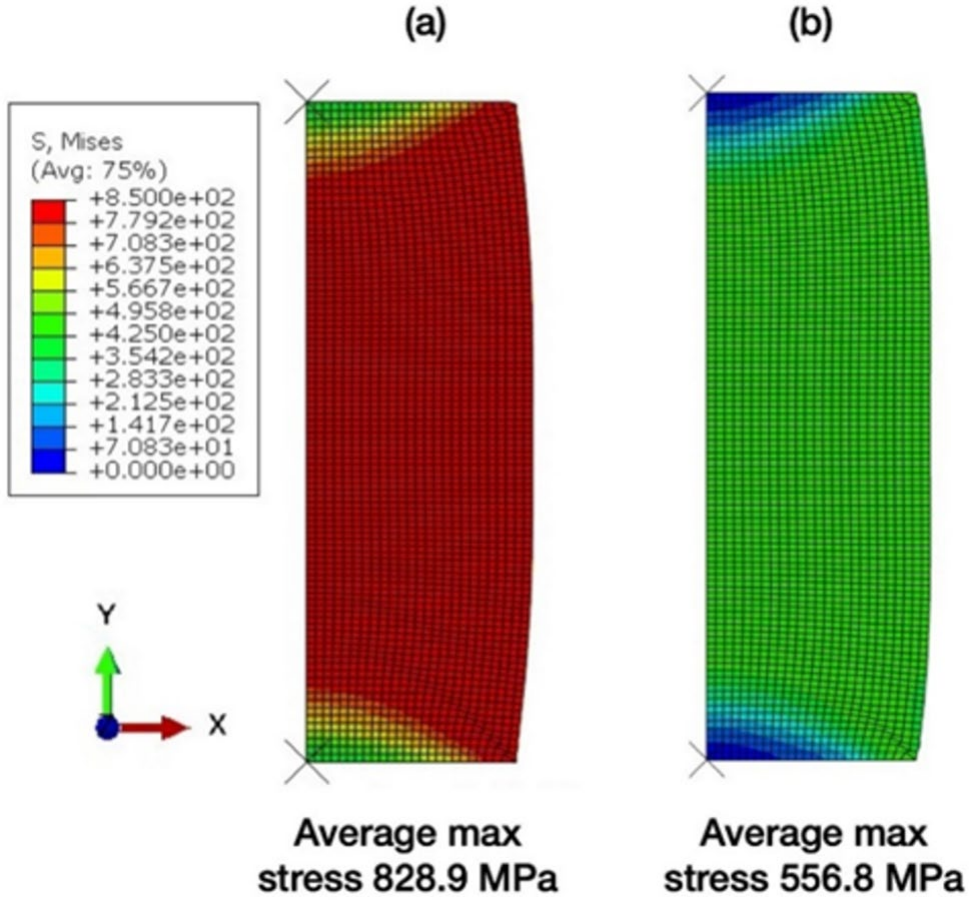
The von Mises stress result of AA7075 compression at (**a**) cryogenic temperature, and (b) room temperature.

**Figure 7:**
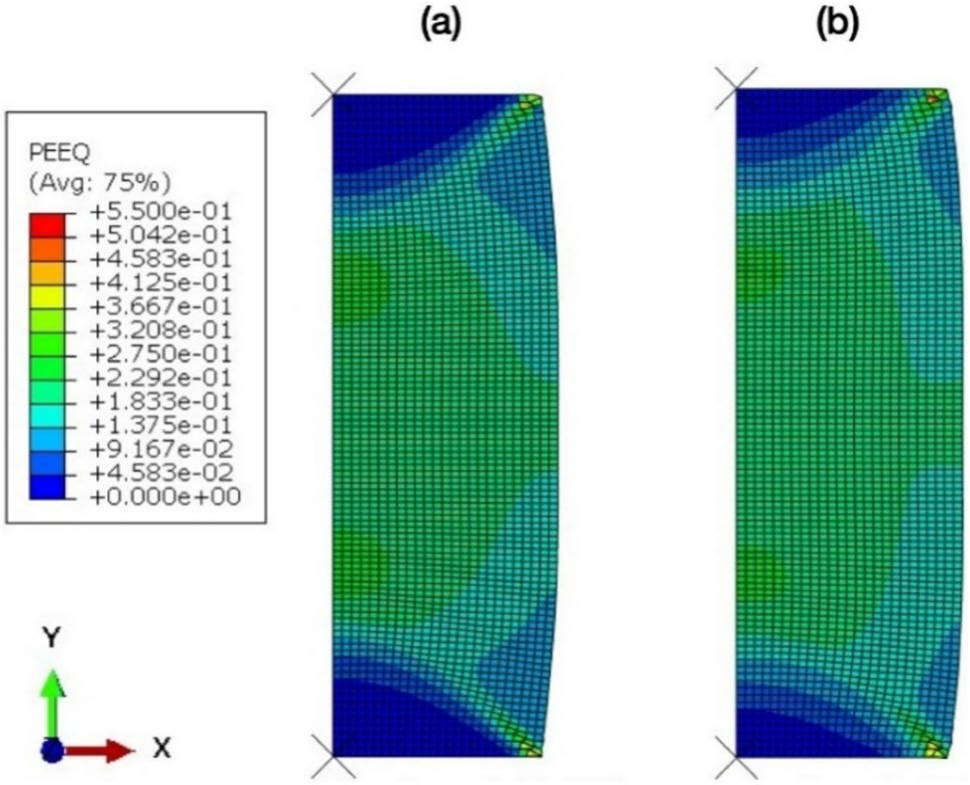
The equivalent plastic strain (PEEQ) result of AA7075 compression at (**a**) cryogenic temperature and (**b**) room temperature.

Figure 5 shows the calculated true stress–strain curves from the finite element model following from the initial step until the final step of the finite element model. The calculated stress–strain responses of the alloy deformed at room temperature and cryogenic temperature agreed well with the experimental results (Fig. 3), as can be seen from the experimental curves added to Fig. 5. The distributions of equivalent local stresses and plastic strains of cast specimens under compression load are illustrated in Figs. 6 and 7, respectively. The residual stress after compression process was evaluated using the von Mises stress. The results showed that the model under cryogenic temperature had a higher average stress than the room temperature model as shown in Fig. 6a and b. The maximum von Mises stress increased from about 556 MPa to 828 MPa, which is located at the red area in the dead metal zone as shown in Fig. 6a. These stresses in the dead metal zone amplified the strain in the center of the specimen, especially for the cryogenic temperature. The equivalent plastic strain (PEEQ) from the list in the ABAQUS output variable was evaluated. The PEEQ represents the inelastic deformation region, which is shown as the positive value in the yielded region. Figure 7a and b demonstrate that the PEEQ of the testing model at cryogenic temperature was higher than that at room temperature.

**Figure 8:**
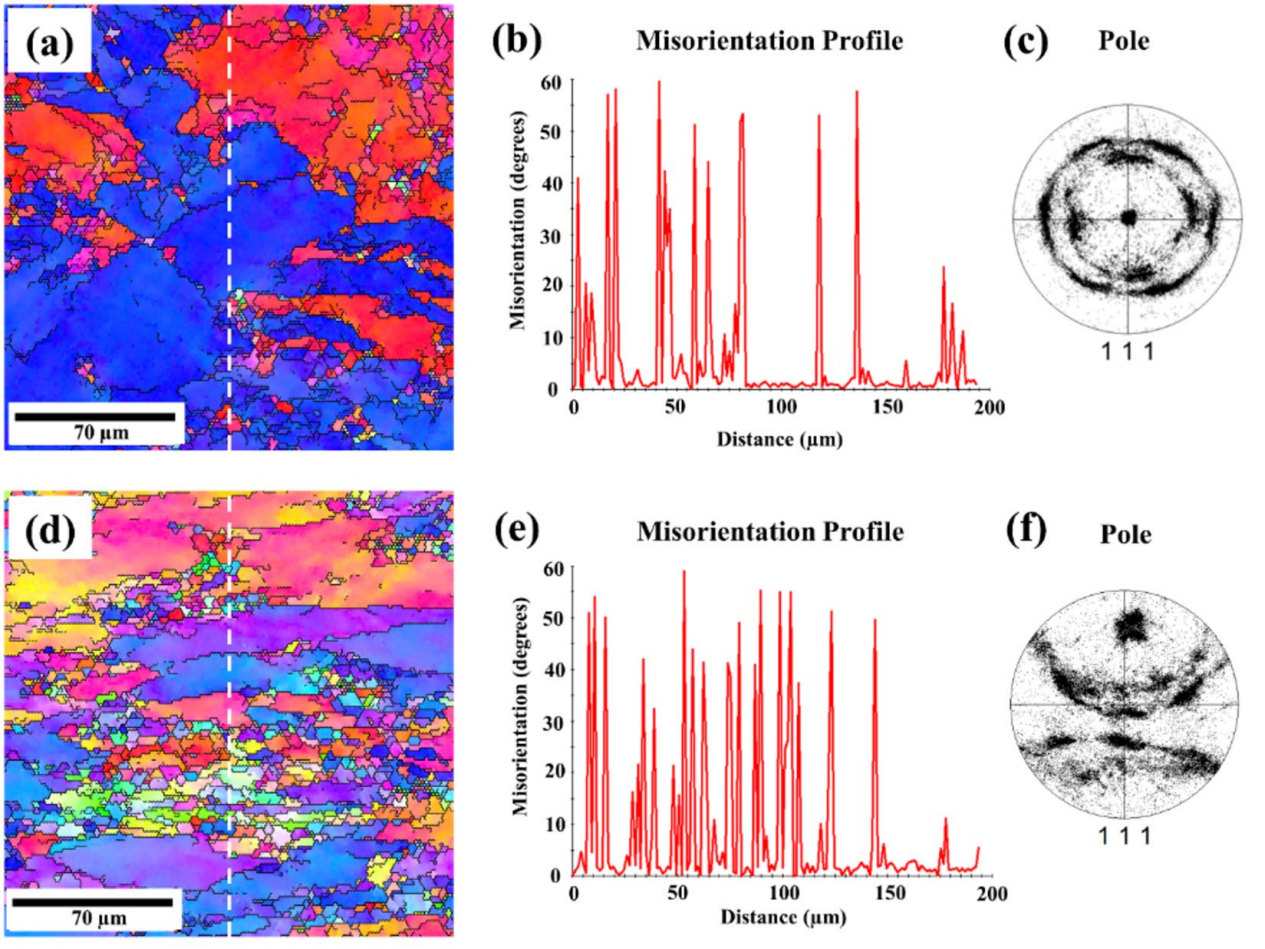
EBSD results with pole image analysis of AA7075 alloy after compression testing at strain rate 0.01 s^−1^ under (**a**, **b**, **c**) room temperature and (**d**, **e**, **f**) cryogenic temperature.

**Figure 9:**
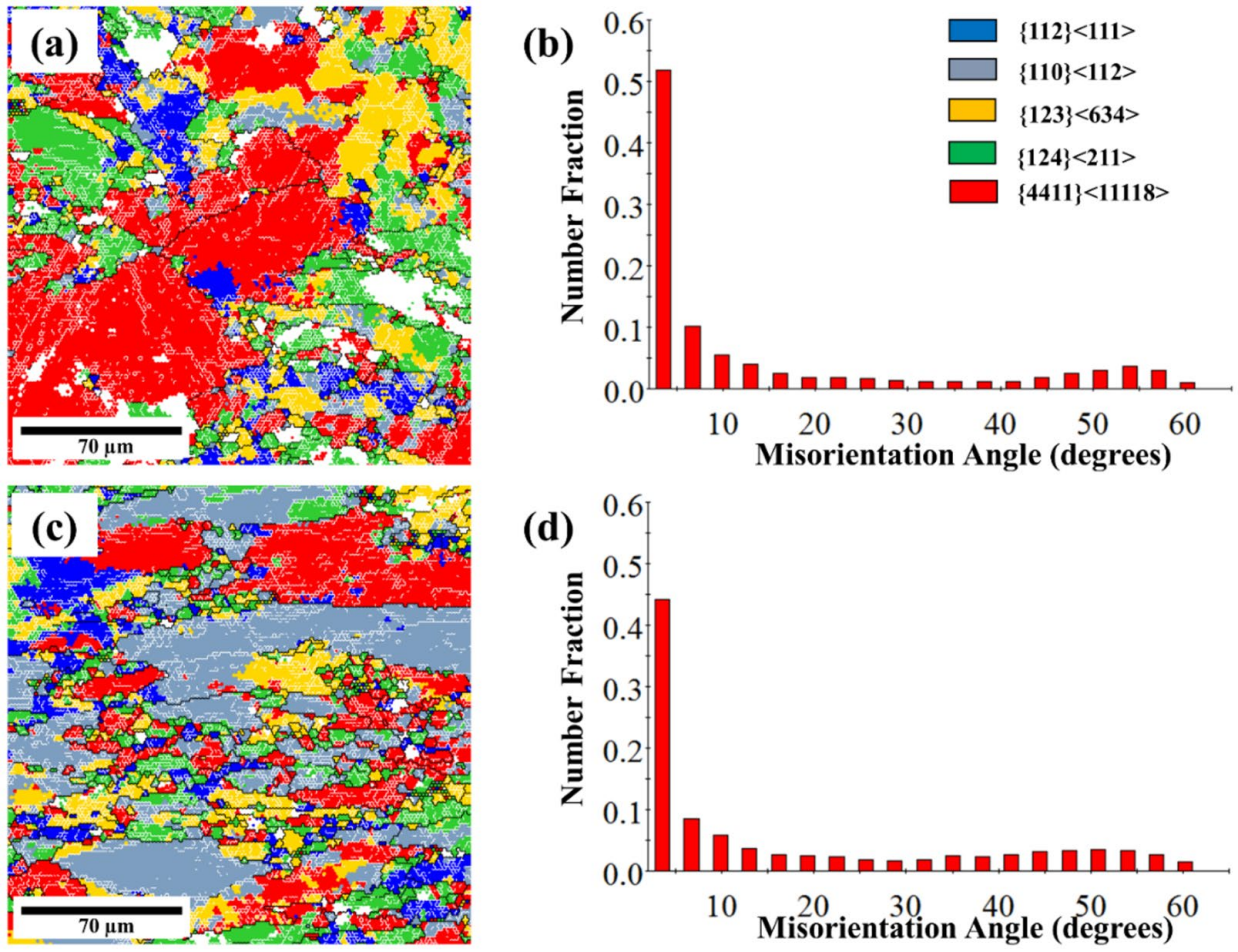
EBSD micrographs and misorientation distribution histograms for the AA7075 alloy in different states: (**a**, **b**) room temperature and (**c**, **d**) cryogenic temperature. (**b**) show misorientation distribution histograms (LAGBs) of (**a**), and (**d**) shows the misorientation distribution histograms (HAGBs) of (**c**). A given color in the micrograph denotes orientations within 15° of an ideal texture component.

The presence of the Al_2_CuMg phase in Fig. 2 can influence the texture evolution and misorientation during deformation, as shown in Figs. 8 and 9. This influence leads to specific texture components^34^, which may affect subsequent processing steps or material properties, such as dislocation accumulation, subgrain formation or recrystallization. These factors, in turn, affect the overall mechanical properties of the alloy, as evidenced by the high higher stress in Figs. 3 and 5^35^. Recrystallization analysis by EBSD is capable of quantifying the crystallographic characteristics of grain boundaries, thus providing better insight into the role of grain boundaries in deformation upon cryogenic treatment. Figure 8 shows the grain structure, inverse pole figure (IPF) orientation maps, misorientation profile and (111) pole figure of the samples deformed at room temperature and cryogenic temperature. It can be seen from Fig. 8d that the elongated grains and fine sub-grains (5–10 µm) with the copper texture were observed in the alloy deformed at cryogenic temperature. Meanwhile, the equiaxed grains were observed in the sample deformed at room temperature as shown in Fig. 8a, and the average grain sizes 50–200 µm was larger than those in the alloys deformed at a low temperature. Figure 8b and e give plots of the change in misorientation along the vertical line in Fig. 8a and d, showing the spacing of the high-angle grain boundaries (HAGBs). It can be seen that the spacing of HAGB in the sample deformed at room temperature is slightly larger than that in the cryogenically treated one, which is indicative of recovery and recrystallization during the forming process. It is confirmed by the misorientation distribution analysis as illustrated in Fig. 9b and d showing a higher fraction of low-angle boundaries (LABs) in the room temperature deformed sample as compared to those deformed at the cryogenic temperature. The high fraction of LABs indicates that most grains were aligned with similar orientations. All the grains or newly formed grains in the temperature deformed sample resulted from recrystallization. In terms of crystallography, the textures were of fiber type. The textures were obtained from inverse pole figures. To simplify the presentation, only the crystallographic direction parallel to the loading axis is presented. Since there was no well-defined rolling direction (RD) and transverse direction (TD) in the compression deformation (unlike rolling), the direction parallel to RD was neglected while representing textures. The EBSD data in Fig. 9 shows a typical fiber texture obtained after compression test. A given color in the micrograph denotes orientations within 15° of an ideal texture component. The room temperature deformed sample tended to show a double fiber texture comprising dominantly < 111 > and < 100 > fibers as shown in Fig. 9a. The crystallographic texture of the cryogenic temperature deformed sample was dominated by a typical deforming texture such a β fiber. Table 3 summarizes the volume fractions of the main textures. According to the results, the S orientation texture (S1 and S3) had the highest volume fraction in the room temperature deformed sample, while the Brass component prevailed in the cryogenic alloy though the S1 + S3 components almost equaled that. The previous research also reported that the volume fraction of the deformed texture components ({112} < 111 > (Copper), {123} < 634 > (S3) and {110} < 112 > (Brass)) in the cryogenic temperature deformed sample were obviously higher than those after room temperature deformation^36^. Meanwhile, the Taylor components for the room temperature deformed sample were higher than those after cryogenic deformation. Therefore, it can be speculated that cryogenic treatment can suppress the recovery and recrystallization during the forming process. This corresponds to the previous work on AA6xxx- and AA2xxx-series alloys that showed that the recrystallization components of these deformed aluminum alloys under cryogenic temperature were lower than those of Al alloys at room temperature^37,38^.

**Table 3:** Volume fractions of main texture components in cryogenic temperature and room temperature (%).

Sample	Deformation texture component				Recrystallization component
	Copper	Brass	S3	S1	Taylor
	{112} < 111 >	{110} < 112 >	{123} < 634 >	{124} < 211 >	{4411} < 11118 >
Cryogenic temperature	10.0	33.4	13.7	15.4	25.7
Room temperature	8.6	6.8	16.7	19.1	40.7

There are two possible phenomena found in cryogenic treated alloys that are related to the refinement of grain and the increase of mechanical properties. In the first phenomenon, cryogenic treatment can produce bulk fine-grained alloys with superior mechanical properties based on the Hall–Petch relation, as confirmed by finite element analysis (Figs. 6 and 7) and the smaller grain as shown in Fig. 9c, which led to higher stress in Figs. 3 and 5. In the second phenomenon, the cryogenic treatment can suppress the recovery and recrystallization during the forming process. Thus, the specimen under cryogenic temperature should have finer grains. The detailed analysis of the grain structure is given in our EBSD results in Figs. 8 and 9 and is in agreement with the results reported elsewhere for other types of Al alloys^37,38^.

## Conclusions

This work investigated the deformation behavior of an AA7075 alloy at room and cryogenic temperatures. The novelty is in the new aspect of cryogenic behavior that would be useful for sheet forming. The main objective of the present research is to understand the responses of the recrystallization and texture of the untreated and cryogenically treated alloys after deformation. The validated finite element simulation allowed us to estimate the residual stress and plastic strain equivalent associated with the microstructure from the experimental alloy. The conclusions drawn from computational and experimental results are as follows:

- The deformability of an AA7075 alloy can be improved by cryogenic deformation with the potential to replace the conventional deformation at room temperature for stamping forming.
- The increased strain hardening rate of AA7075 alloy deformed at cryogenic temperatures can linked with a change in the dynamic recovery.
- The experimentally found increase in the flow stress corresponded to the FE results that showed high stress and PEEQ after the alloy was deformed at cryogenic temperature, due to the suppressed recovery and recrystallization during the cryogenic forming.
- The tested aluminum alloy showed more elongated grains and fine sub-grains after being deformed at the cryogenic temperature, due to the hindered recrystallization.

## Data Availability

The data that support the findings of this study are available from the corresponding author upon reasonable request.
